# Role of Heme Oxygenase in Inflammation, Insulin-Signalling, Diabetes and Obesity

**DOI:** 10.1155/2010/359732

**Published:** 2010-05-18

**Authors:** Joseph Fomusi Ndisang

**Affiliations:** Department of Physiology, College of Medicine, University of Saskatchewan, 107 Wiggins Road, Saskatoon, SK, Canada S7N 5E5

## Abstract

Diabetes and obesity are chronic conditions associated with elevated oxidative/inflammatory activities with a continuum of tissue insults leading to more severe cardiometabolic and renal complications including myocardial infarction and end-stage-renal damage. A common denominator of these chronic conditions is the enhanced the levels of cytokines like tumour necrosis factor-alpha (TNF-*α*), interleukin (IL-6), IL-1*β* and resistin, which in turn activates the c-Jun-N-terminal kinase (JNK) and NF-*κ*B pathways, creating a vicious cycle that exacerbates insulin resistance, type-2 diabetes and related complications. Emerging evidence indicates that heme oxygenase (HO) inducers are endowed with potent anti-diabetic and insulin sensitizing effects besides their ability to suppress immune/inflammatory response. Importantly, the HO system abates inflammation through several mechanisms including the suppression of macrophage-infiltration and abrogation of oxidative/inflammatory transcription factors like NF-*κ*B, JNK and activating protein-1. This review highlights the mechanisms by which the HO system potentiates insulin signalling, with particular emphasis on HO-mediated suppression of oxidative and inflammatory insults. The HO system could be explored in the search for novel remedies against cardiometabolic diseases and their complications.

## 1. Background

There has been a dramatic rise in the number of patients with the metabolic syndrome, a comorbid condition of hypertension, obesity, and diabetes. Diabetes mellitus is a chronic syndrome of impaired carbohydrate, protein, and fat metabolism caused by insufficient secretion of insulin and/or defects in insulin action in tissues due to insulin resistance. The incidence of diabetes is increasing globally [1] and type-2 diabetes (TD2) accounts for almost 90% of the cases diagnosed [2–4]. It is projected that the prevalence of T2D may reach 366 million in 2030 [1]. Similarly, the condition of obesity has escalated as more than 300 million adults, the majority of whom live in the developed world, are affected [5]. Obesity is amongst the main risk factor for insulin resistant T2D, hypertension, and other cardiovascular and renal complications [6]. Although inadequate insulin production is traditionally linked to type-1 diabetes (T1D), emerging evidence suggests that pancreatic beta-cell mass is reduced during the early stages of T2D and declines further with the progression of disease, eventually leading to loss of beta cells and reduced insulin production [7, 8]. This is consistent with previous observation indicating that T2D is not solely due to insulin resistance but also due to a failure of the insulin producing beta-cells to secrete an adequate amount of insulin [9]. On the other hand, in T1D it is a well-established concept that genetic defects trigger autoimmunity leading to the destruction of pancreatic beta cells and insulin insufficiency [10], and these events are further accentuated by apoptosis [11–13]. Similarly, in T2D, intense inflammatory activities characterized by the presence of cytokines, apoptotic cells, immune cell infiltration, amyloid deposits, and fibrosis may cause reduction of pancreatic beta-cell mass [14]. In both T1D and T2D, elevated inflammatory events play a major pathophysiological role in the disruption of islet architecture [10, 14–20]. Several factors are responsible for inflammation in T1D and T2D. These include dyslipidemia, hyperglycaemia, elevated nuclear-factor kappaB (NF-*κ*B) activity, increased levels of adipokines such as tumour necrosis factor-alpha (TNF*α*), interleukins (ILs), resistin, leptin and free fatty acids [14, 21]. Seen in this light, the suppression of apoptosis, necrosis, and intraislet inflammatory/immune events may be important for the preservation of islet architecture and beta-cell morphology. Therefore, the regulation of beta-cell number through the processes of proliferation, neogenesis, and apoptosis is important to safeguard islet function [22, 23] and the maintenance of adequate insulin production in T1D and T2D. Taken together, these studies suggest that impaired insulin secretion is not only an important etiological factor in the pathogenesis of T1D and T2D, but also an important pathophysiological driving force that is capable of dictating the dynamics and progression of the disease. Thus novel therapeutic modalities capable of suppressing inflammatory/immune responses, apoptosis, and necrosis would be beneficial in the conditions of T1D and T2D. 

Generally, insulin resistance and T2D frequently occur in obesity [24–35]. Amongst the contributing factors, are overnutrition and inactivity. As an adaptive response to insulin resistance, pancreatic islets enhance their secretory activity. In most individuals, such an adaptation does occur during early stages of overnutrition and metabolism would appear normal at this stage. However, at later stages, this adaptation eventually fails in some individuals, depending on the genetic ability of the beta-cell to adapt and the severity of the resistance to insulin [36]. The reasons for this failure to maintain sufficient insulin secretion are a combined decrease in beta-cell mass and insufficient secretion of insulin. This reduction of insulin levels may be due to elevate inflammation, oxidative stress, amyloid deposition, lipotoxicity, and glucotoxicity [36]. Obesity and insulin resistance are associated with a state of low-grade inflammation due to chronic activation of innate immune system [37]. Although epidemiological studies have linked inflammation with obesity for decades, the underlying mechanisms remained obscured until the last decade. It is now widely accepted that the activation of inflammatory mediators such as NF-*κ*B, TNF*α*, and c-Jun-N-terminal kinase (JNK) is amongst the common causes of insulin resistant T2D in obsessed conditions [24–35]. Thus, novel strategies that can preserve beta-cell integrity improve insulin sensitivity, and counteract inflammatory mediators like NF-*κ*B, TNF-*α*, and JNK would be useful in the prevention and management of insulin resistant T2D and related cardiometabolic complications. Recent evidence has highlighted the important role of the heme oxygenase (HO) in insulin release and glucose metabolism [38–52]. Beside its emerging antidiabetic effects, the HO system is also known to abate oxidative stress and immune/inflammatory response [53–57]. This review will highlight the mechanisms by which the HO system potentiates insulin signalling, with particular emphasis on HO-mediated suppression of inflammation.

## 2. The HO System and Insulin Signaling

**Figure 1:**
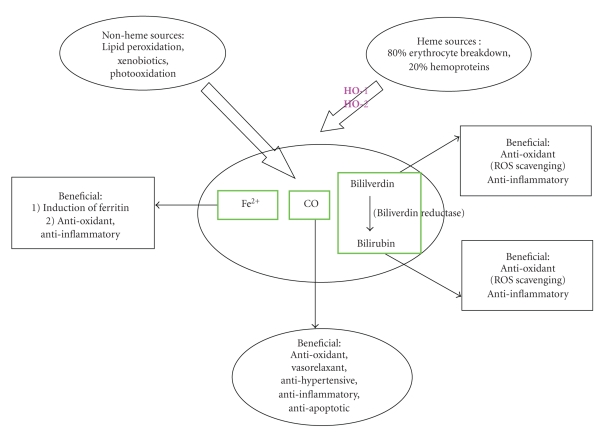
In the human body, carbon monoxide is formed at a rate of 16.4 *μ*mol/h and daily production can reach 500 *μ*M (Piantadosi, *Antioxid Redox Signal, *2002, 4:259-70). About 86% comes from HO-catalyzed degradation of heme while 14% from lopid peroxidation xenobiotics and other sources.

HO is a microsomal enzyme that cleaves the *α*-methene bridge of heme moiety to produce equimolar amounts of carbon monoxide (CO), bilirubin, and iron [58, 59] (Figure 1). CO and bilirubin are known to suppress apoptosis, necrosis, inflammation, and oxidative stress [56, 60–69], while the iron formed enhances the synthesis of the antioxidant, ferritin [70, 71]. 

**Figure 2:**
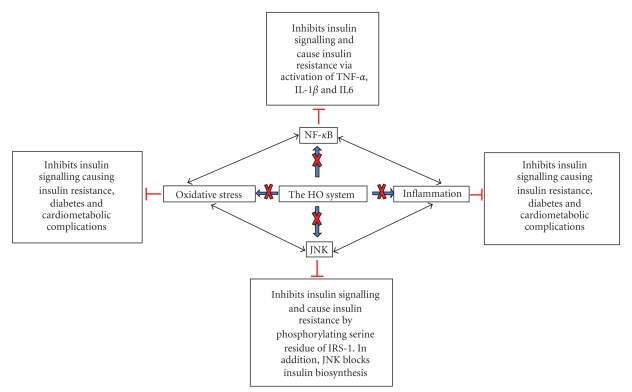
Schematic representation illustrating the protective role of the HO system in glucose metabolism. Inflammatory and oxidative mediators like NF-*κ*B, JNK, TGF-*α*, IL1*β* and IL-6 are amongst the pathophysiological factors that impair insulin signalling. Generally these substances stimulate oxidative/inflammatory events destroying tissue. Conversely, other factors including cytokines and inflammatory/oxidative transcription factors like NF-*κ*B, JNK stimulate a variety of different pathophysiological pathways to further aggravate oxidative/inflammatory insult, creating a vicious cycle of intense inflammation that would severely damage tissue and compromise many physiological functions including glucose metabolism. However, the HO system suppresses these inflammatory/oxidative mediators and pro-inflammatory cytokines to enhance insulin signalling and improve glucose metabolism.

The main isoforms of HO include HO-1 (inducible) and HO-2 (constitutive) isoforms [58, 59, 72, 73]. HO-1 and HO-2 are largely responsible for HO enzymatic activity [58, 72, 73], while the third isoform, HO-3, has no functional genes in rat and is considered a pseudotranscripts of HO-2 [74, 75]. The basal HO activity is maintained by HO-2 [58, 59, 72, 73, 76], while HO-1 is stimulated by a wide variety of different physical, chemical, and pathophysiological stimuli including oxidative and inflammatory insults [58, 59, 77–80], as well as metabolic and hemodynamic factors such as high glucose [80], elevated blood pressure [64], and lipids [81]. Therefore, HO-1 may be considered a sensitive index that is triggered in the onset of pathophysiological changes. However, in most cases the pathophysiological activation of HO-1 results only to a transient or marginal increase of HO-1 that falls below the threshold necessary to activate the downstream signalling components of the HO system [59, 63, 82]. For example, the pathophysiological activation of HO-1 by the hemodynamic stress of elevated blood pressure is not accompanied by changes of important component of HO-signalling like cyclic guanosine monophosphate (cGMP) [59, 63, 82–85]. Therefore the transient upregulation of HO-1 that normally accompanies many pathophysiological conditions may represent the first line of defense mounted against tissue injury to counteract adverse changes that would destabilize the homeostatic conditions in physiological milieu. Since the pathophysiological activation of HO-1 may fall below the threshold necessary to activate important signalling components through which the HO system elicits its effects of restoring tissue homeostasis [63, 82], a more robust enhancement of HO-1 would be needed to surmount the threshold [63, 82–85]. This can be achieved by pharmacological agents capable of inducing HO like some metalloprotoporphyrin such as hemin (ferric protoporphyrin IX chloride), stannous mesoporphyrin, copper protoporphyrin, and cobalt protoporphyrin. Given that many of the adverse factors which stimulate HO-1 such as elevated blood pressure [64] and high glucose and lipid [80, 81] concentrations are implicated in the pathophysiology of metabolic syndrome, the HO system may constitute a novel approach that could be explored against metabolic syndrome and related cardiometabolic complications (Figure 2). 

The emerging role of the HO system in insulin release and glucose metabolism is becoming increasingly clear [38–52]. HO-mediated stimulation of insulin release has been reported in different rats strains [38, 46, 49–52] and mice [86, 87]. These studies suggest a central role of CO in glucose metabolism. In the human body, CO is formed at a rate of 16.4 *μ*mol/h and daily production of may reach 500 *μ*mole [88]. Interestingly, under normal physiological conditions, islets of Langerhans produce CO and nitric oxide (NO) to regulate insulin release [45, 46]. While NO negatively modulates glucose-stimulated insulin release, CO stimulates insulin secretion [45, 46]. Moreover, glucose stimulates pancreatic beta-cells to produce CO, which in turn triggers insulin release [45, 46]. The critical role of the HO system in insulin release and glucose metabolism was reported in Goto-Kakizaki (GK) rats, a model with defective pancreatic beta-cell HO-2 [38]. Since HO-2 is largely responsible for basal HO activity [58, 59, 72, 73, 76] and thus the production CO, the impairment of the HO system in GK rats resulted in reduced CO and insulin insufficiency [38]. Interestingly, treatment with the HO-inducer, hemin, or CO corrected the defective HO system and enhanced insulin release with improvement of glucose metabolism [38]. Collectively, these studies suggest that reduced beta-cell CO and/or impaired HO system may lead to dysfunctional glucose metabolism.

## 3. The Role of HO System in Inflammation and Insulin Resistance

The inflammatory and metabolic systems are among the most fundamental for survival, and these systems have been evolutionarily well-conserved in species [37]. However, the conditions of nutrient-overload or obesity may offset these systems leading to inflammation in metabolic sites like the adipose tissue, liver, and skeletal muscles. One consequence of such imbalance is the increased production of proinflammatory cytokines, adipokines, and other inflammatory/oxidative transcription factors including NF-*κ*B activating protein (AP-1) and JNK. Although both JNK and NF-*κ*B play important roles in inflammation-induced insulin resistance, accumulated evidence suggests that they do so through different mechanisms. The principal mechanism by which JNK causes insulin resistance is through the phosphorylation of serine residues in insulin receptor substrate-1 (IRS-1) [89–91]. However, since JNK is a stress kinase that also phosphorylates the c-Jun component of the AP-1 [92], the activation of AP-1 by JNK may contribute to aggravate inflammatory insults and hence insulin resistance. NF-*κ*B causes insulin resistance by stimulating proinflammatory cytokines like TNF-*α*, IL-6, IL-1*β*, and resistin, which in turn activates JNK and NF-*κ*B pathways to create a vicious cycle that will exacerbate tissue damage [89, 91, 93–97]. 

 An important trigger of NF-*κ*B, AP-1, and JNK is the renin-angiotensin-aldosterone system (RAS). Like angiotensin-II, aldosterone stimulates inflammation and fibrosis by activating transcription factors such as NF-*κ*B, AP-1, and JNK [98, 99]. Moreover, oxidative stress will further enhance the activation of JNK [100]. On the other hand, JNK blocks insulin biosynthesis [100] and regulates AP-1 [101]. These transcription factors modify insulin signaling and thus are involved in the development of insulin resistance. Therefore, the reduction of oxidative/inflammatory transcription factors in T2D would not only limit tissue insults but also decrease the oxidative destruction of a wide variety of important metabolic regulators including adiponectin and insulin [100, 102]. Therefore, novel therapeutic strategies that concomitantly ablate inflammation and insulin resistanc, but enhance adiponectin are needed. Interestingly, the HO system has been shown to modulate both the metabolic and inflammatory systems suppressing insulin resistance and inflammation while enhancing adiponectin levels [40–44, 47, 48, 51, 55, 56, 82, 103–113]. Therefore the inflammatory and metabolic effects of HO may be highly integrated and the proper function of each may depend on the other [37]. Given that insulin resistance may trigger inflammatory events [114], it remains to be clarified whether insulin resistance precedes the development of inflammation or vice versa. Further investigation in this regard will advance our knowledge in the development of more specific therapeutic modalities.

Adiponectin is a cytoprotective protein produced by the adipose tissue. It is composed of several multimeric species or isoforms with low-, middle-, or high-molecular weights [115]. The high-molecular-weight isoform is thought to be the most clinically relevant. Generally adiponectin elicits its effects through its receptors (adiponectin receptor-1 and -2) which, besides activating adenosine monophosphate protein kinase (AMPK), also activates peroxisome proliferator-activated receptor alpha (PPAR*α*) in the liver to increase insulin sensitivity and decrease inflammation [116–118]. Generally, the high-molecular weight adiponectin plays a crucial role in obesity-linked insulin resistance and metabolic syndrome. Interestingly, PPAR*γ* upregulates high-molecular weight adiponectin to enhance insulin sensitivity and glucose metabolism [117, 119, 120]. Besides its insulin-sensitizing effect, adionectin has also protective effects against atherosclerosis [121] and inflammation [122]. Moreover, clinical evidence indicates that adiponectin levels are low in patients with obesity, atherosclerosis, and insulin resistance [119]. Furthermore, knocking-out adiponectin leads to insulin-resistant T2D [120]. Collectively, these studies underscore the important role of adiponectin in cytoprotection, insulin sensitivity, and glucose metabolism. Insulin insensitivity is a hallmark of T2D [123, 124] the causes include excessive NF-*κ*B activity [125–129], elevated JNK activation [100] and increased production of adipokines including free fatty acids, TNF*α*, ILs, resistin, leptin by the adipose tissue [130–133]. In T2D diabetic patients, insulin resistance may lead to metabolic syndrome, a pathological condition with hyperinsulinemia, hypertension, glucose intolerance, and dyslipidemia [122, 134, 135]. 

We recently showed that the HO inducer hemin is endowed with potent antihypertensive and antidiabetic effects. Interestingly hemin therapy is effective against T1D and T2D. Our findings showed that upregulating the HO system with hemin reduced fasting and postprandial hyperglycaemia in different insulin-resistant T2D models, including nonobese Goto-Kakizaki rats (GK) [42, 44] and Zucker diabetic fatty rats (ZDF) [43], a genetically obese leptin receptor-deficient (fa/fa) model [136, 137]. Interestingly, after termination of therapy, the antidiabetic effects prevailed for 3 and 4 months, respectively, in GK and ZDF [42–44]. Further revelations from our findings indicate that hemin therapy is also effective against streptozotocin- (STZ-) induced diabetes [41] and improves insulin sensitivity/glucose metabolism in spontaneously hypertensive rats (SHRs) [47], a model of essential hypertension and with features of metabolic syndrome like insulin resistance and impaired glucose metabolism [138, 139]. Furthermore we showed that hemin improved insulin-signaling/glucose metabolism in deoxycorticosterone-acetate (DOCA) hypertension, a model of primary aldosteronism [48], suggesting a role of the HO system against dysfunctional glucose metabolism in aldosteronism. Interestingly, the antidiabetic effect of hemin was accompanied by a paradoxical increase of plasma insulin and enhanced insulin-sensitivity [41–44], alongside the potentiation of agents that promote insulin-signalling, including adiponectin [40–44, 47, 48, 108–113] cGMP [45, 140], cyclic adenosine monophosphate (cAMP) [45], adenosine monophosphate-activated protein-kinase (AMPK) [141, 142], aldolase-B [143], and glucose-transporter-4 (GLUT4) expression [142, 144]. Correspondingly, hemin improved intraperitoneal glucose-tolerance (IPGTT), reduced insulin-tolerance (IPITT), and lowered insulin resistance (HOMA index), and the inability of insulin to enhance GLUT4 was overturned [41–44]. Furthermore, hemin therapy potentiated the antioxidant status in ZDF, GK, and STZ-diabetic rats with the suppression of oxidative/inflammatory mediators including 8-isoprostane, NF-*κ*B, AP-1, AP-2, and JNK [41–44]. 

Given that diabetes is characterized by elevated oxidative and inflammatory insults, the HO system would suppress these insults by generating CO, bilirubin/biliverdin and ferritin against apoptosis, inflammation and oxidative stress [66–68, 71, 145–147]. Thus, the insulin-sensitizing effects of hemin, when combined to its antihypertensive effects [58, 59, 63–65, 83–85, 148–154], underscores the important role of the HO system that could be explored against impaired glucose metabolism and hypertension given the rising incidence of comorbidities of essential hypertension, glucose intolerance, and insulin resistance [155, 156] as well as pathophysiological conditions like primary aldosteronism, glucose intolerance, and insulin resistance [157–159]. 

### 3.1. The HO System, NF-*κ*B, and Inflammation

The HO-1 promoter harbours consensus binding sites for many substances including inflammatory/oxidative transcription factors like NF-*κ*B, AP-1, and AP-2 as well as motifs for glucocorticoid-responsive elements [160, 161]. As such, the HO system may regulate inflammation and insulin release [41–44, 47, 48, 162]. Given that HO-1 is induced by different stimuli including high glucose levels [77, 80], the diversity of HO inducers may be indicative of multiple regulatory elements for the HO-1 gene with binding sites for different transcription factors or genes. These arrays of genes may account for the diverse and pleitropic effects of the HO system in many cellular events including defence and glucose metabolism [40–44, 47, 48, 65, 163–166]. By modulating a wide variety of transcription factors, cellular metabolism may be regulated. Thus, the HO system may be crucial for the coordination and proper functioning of basic physiological units in animals. More importantly, the regulation of NF-*κ*B by HO-1 may be important for cellular homeostasis given the pleitropic effects of NF-*κ*B-signalling in many pathophysiological conditions including inflammation and insulin resistance [125–129] (Figure 2). 

Transcription factors are proteins that act as a sensor to monitor cellular change and convert the signals into gene expression. Generally, a specific cellular signal pathway can activate multiple transcription factors and the expression of a specific gene may be controlled by multiple transcription factors. Importantly, transcription factors mediate signal transduction by binding to specific DNA sequence in gene promoters to regulate transcription activity. Although the exact characterization of the series of events and the mechanisms that integrate the inflammatory response with metabolic homeostasis at the cellular and systemic level are not fully understood, emerging data indicates that NF-*κ*B plays a key role [125, 127, 128, 167–169]. NF-*κ*B is a family of transcription factors that generally function as heterodimers to regulate specific gene expression. In the quiescent state, NF-*κ*B is trapped in the cytoplasm by its interaction with the inhibitory protein, “inhibitor of NF-*κ*B kinase subunit beta” (IKK*β*). The IKK*β*/NF-*κ*B complex is an essential mediator of inflammatory cascades. Importantly, the IKK*β*/NF-*κ*B complex plays a critical and fundamental role for immunity and survival [125, 167]. The proteosomal degradation of the IKK*β*/NF-*κ*B complex is triggered by different stimuli or pathophysiological conditions. Upon activation by stimuli like oxidative stress, lipopolysaccharide endotoxin (LPS), mitogens, or cytokines, the phosphorylation of Ser177 and Ser181 activates the complex, triggering a cascade of reactions that leads to proteolysis of IKK*β*-specific protein kinase and the release of the NF-*κ*B. Upon release, NF-*κ*B translocates into the cell nucleus where it mediates the transcriptional activity of a wide variety of target genes [170–172]. The transcriptional products of NF-*κ*B in immune cells include different cytokines and their receptors, adhesion molecules, immune modulators, and apoptotic factors, all of which are implicated at various stages during the inflammatory cascade.

Besides its traditional role in the immune/inflammation system, emerging evidence suggests that NF-*κ*B also mediates chronic low-grade metabolic inflammation in a variety of different tissues including adipose [128], liver [168], and skeletal muscle [127, 169]. Therefore NF-*κ*B can interfere with several molecular programs to cause the different aspects of metabolic dysfunction, especially under chronic conditions like hypertension, diabetes, and obesity or nutritional excess. For example, the NF-*κ*B has been linked to insulin resistance and numerous physiological deregulations that underlie overnutrition [125–129]. Generally, insulin resistant T2D is associated with the chronic activation of NF-*κ*B pathway and elevated inflammation [126, 173, 174]. 

A commonly used strategy to alleviate tissue insults and restore cellular metabolism in conditions of elevated inflammation and insulin resistance is PPAR*γ* agonists [175]. PPAR*γ* agonists are a class of drugs used against insulin resistance and T2D [175]. PPAR*γ* is a genetic sensor of fatty acids and a member of the nuclear receptor superfamily of ligand-dependent transcription factors. PPAR*γ* is required for fat cell development and is the molecular target of thiazolidines, a class of insulin-sensitizing drugs that exert their effects in adipose tissue and skeletal muscle [175]. Although a variety of PPAR*γ* agonists are available [175], novel pharmacological agents would be needed in the therapeutic armament giving the recent escalation of insulin resistant T2D, metabolic syndrome, and cardiometabolic complications. 

We recently showed that upregulating the HO system with hemin suppressed NF-*κ*B in different models of T2D including ZDF and GK rats [42–44], as well as different hypertensive models including SHR [47, 84] and DOCA-hypertensive rats [48, 65, 84, 150, 151]. Similarly, other HO inducers has been shown to be effective against insulin resistant T2D [39, 40, 46, 49, 111, 113, 176]. Therefore, HO inducers may be explored in the design of novel strategies against insulin resistant diabetes. Incidentally, PPAR*γ* have been shown to upregulate high-molecular weight adiponectin [117, 119, 120], an insulin-sensitizing agent. Similarly, adiponectin is upregulated by the HO system [40–44, 47, 48, 108–113]. Therefore the synergistic effects of PPAR*γ* and the HO system in improving insulin sensitivity and glucose metabolism may be a novel approach to combat insulin resistance and related cardiometabolic complications.

### 3.2. The HO System, cJNK, and Inflammation

JNK proteins belong to the mitogen activated protein kinase family and control transcriptional activity of AP-1 via phosphorylation of c-Jun [92]. Three closely related JNK isoforms including JNK1, JNK2, and JNK3 have been described. Generally, JNK-signalling is activated by inflammatory cytokines and environmental stressors [177]. Reports indicate that the different JNK isoforms are implicated in a wide variety of pathophysiological conditions caused by inflammatory insults. These include insulin resistance, T2D, infectious diseases, stroke, Parkinson's disease, and cardiovascular disorders [92]. The tissue distribution and activities of JNK1, JNK2 and JNK3 isoforms are different. JNK1 and JNK2, are widely expressed in tissues and are involved in different activities including T-cell activation and brain development [92]. On the contrary, JNK3 is less-diffused and is predominantly expressed in neurons in the hippocampus and mediates neuronal apoptosis. 

In obesity, JNK activity is increased in the liver, muscle, and fat tissues probably due to the increase of free fatty acids and TNF-*α* [92, 177]. Interestingly, JNKs are key signalling molecules that link inflammation and insulin resistance (Figure 2). The role of JNK in insulin resistance is highlighted in studies showing that the abrogation of JNK prevents insulin resistance in obese and diabetic mice [178–180]. In contrast, overexpression of a dominant-negative proteins for JNKs or knocking down JNK1 by RNA interference assay resulted in the inhibition of JNK with improved insulin sensitivity [178–180]. Similarly, genetic disruption of JNK1 gene reportedly prevented the development of insulin resistance in obese and diabetic mice [181]. Moreover, under diabetic conditions, oxidative stress activates JNK, which in turn suppresses insulin biosynthesis [100] causing impaired insulin-signalling and glucose metabolism. Conversely, the suppression of JNK resulted in reduced insulin resistance and improved glucose tolerance in diabetic mice [100]. 

The role of JNK in insulin resistance has been further highlighted by its interaction with IRS-1. An important step during the insulin-signal transduction cascade is the activation of insulin receptor tyrosine kinase and the resulting phosphorylation of IRS-1. Subsequently, through the activation of phosphatidylinositol 3-phosphate kinase (PI3K), insulin regulates different metabolic pathways. These include the activation of glucose uptake in muscle and fat, downregulation of gluconeogenesis in liver, upregulation of glycogen synthesis, and induction of protein synthesis. However, these important insulin-mediated signalling events could be halted if serine of the IRS-1 is phosphorylated instead of tyrosine. Several stress-related kinases, including JNK, induce the serine phosphorylation of IRS-1 and thus inhibit the insulin-signal transduction cascade. Interestingly, JNK-mediated phosphorylation of serine is a common pathophysiological event in obesity [90, 91]. In a related study, obesity-induced stress was shown to cause insulin resistance via JNK-mediated phosphorylation of inhibitory serine residues IRS-1 [90, 91]. Collectively, these studies underscore the important role of JNK in insulin resistance and suggest that inhibitors of JNK-signalling may be used as insulin sensitizing agents. Thus, the genetic ablation of one or more JNK isoforms may be a novel strategy against insulin resistant T2D and related obesity-induced cardiometabolic complications. 

A number of different pharmacological agents capable of inhibiting JNK are presently under investigations. These include different classes of inhibitors: small-molecule JNK inhibitors which may be derivatives of anthrapyrazolone, imidazoles, anilinoindazole, pyrazoloquinolinones, aminopyridines, or pyridine carboxamide [182, 183]. Other classes of compounds under studies are ATP-competitive JNK inhibitors and peptide substrate-competitive ATP-noncompetitive JNK inhibitors [182, 183]. These include diaryl-imidazoles, anilinoindazoles, pyrazoloquinolinones, aminopyridines, pyridine carboxamides, anilino-bipyridines, and anilino-pyrimidines and compound SP600125 [182, 183]. Although these compounds are promising as they are endowed with good potency and greater selectivity, their practical application in clinics is a long way ahead; so other alternative modalities to block JNK-signalling would be useful. Interestingly, we recently showed that upregulation of the HO system with hemin suppressed JNK and improved insulin sensitivity and glucose metabolism in STZ-induced diabetes, insulin resistant T2D models like ZDF and GK; as well as in hypertensive models like SHR and uinnephrectomised DOCA-salt rats [41–44, 47, 48]. The attenuation of JNK by hemin was consistent with previous reports in which an upregulated HO system reportedly abrogated JNK [184]. Although significant contributions have been made in delineating the role of JNK and its isoforms in cardiometabolic complications, further studies are needed to identify more specific inhibitors and/or novel compounds with improved pharmacokinetics and pharmacodynamics.

### 3.3. The HO System and Obesity and Inflammation

Obesity and insulin resistance are pathophysiological cardinal features of metabolic syndrome. Generally, obesity and insulin resistance are closely associated with a state of low-grade inflammation of white adipose tissue as a result of chronic activation of the innate immune system leading to impaired glucose tolerance, diabetes and other cadiometabolic complications [37]. Although epidemiological studies had linked inflammation with obesity for decades, the underlying mechanisms remained obscured until the last decade when strong evidence indicated that obesity is a condition associated with chronic inflammatory activity due to incessant activation of a wide variety of inflammatory mediators including NF-*κ*B, TNF-*α* and JNK [25–35]. Similarly, free fatty acids binding innate immune receptors like Toll-like receptor (TLR4) have been shown to trigger significant inflammatory activities in the condition of obesity. Consistent with this notion are reports indicating that in TLR4-knockout mice, diet-induced obesity and inflammation is abrogated [185]. On the other hand, the binding of free fatty acids to TLR4 activates the IKK*β*/NF-*κ*B complex and the JNK pathway to initiate a cascade of other inflammatory and proinflammatory factors [186]. Therefore, the secretion of proinflammatory factors by the adipose tissue and the regulation of these secretions by increasing adiposity sustain the notion of an ongoing low-grade inflammatory process in obesity. Emerging evidence indicates that adipocytes from different body compartments have distinct inflammatory phenotype based on their anatomical location and genetic differences between intraabdominal visceral-fat and peripheral subcutaneous-fat [187]. Importantly, visceral adiposity is more malignant than subcutaneous adiposity. These differences are reflected in the contrasting roles of visceral and subcutaneous adiposity in the pathogenesis of obesity-related cardiometabolic complications like insulin resistant T2D and coronary artery disease in lean and obese individuals [187]. Generally, resident macrophages in visceral adipose tissue generate higher levels of proinflamatory cytokines like TNF-*α* and IL6, but reduced levels of the anti-inflammatory adipokine, adiponectin [187]. Changes in the levels of these cytokines are amongst the fundamental causes of inducing insulin resistance and play a major role in the pathogenesis of endothelial dysfunction, T2D, and related cardiometabolic complications like atherosclerosis, especially in the condition of obesity.

In the adipose tissue chronic overnutrition leads to macrophage infiltration, resulting in local inflammation that potentiates insulin resistance. Both TNF-*α* and JNK are implicated in inflammation-induced impairment of insulin signalling in obesity [25–31]. Moreover, NF-*κ*B is a stimulator of TNF*α* [91, 93–97]. The role of NF-*κ*B in inflammation in obesity was demonstrated experimentally in metabolic tissue, by nutrient overload [32, 33]. Accordingly, glucose overload was shown to activate NF-*κ*B in the adipose [128], endothelial, and pancreatic tissues [188–190]. Similarly, lipid overload increased NF-*κ*B activity in humans and animals [128, 191]. Moreover, in cultured cells, tissues and whole animals, NF-*κ*B has been shown to activate TNF*α*, IL6, IL-1*β*, and plasminogen activator inhibitor 1 (PAI-1) inducing insulin resistance [91, 93–97]. Collectively, these studies strongly suggest a role of the NF-*κ*B pathway in nutrition-overload induced insulin resistance and its involvement in aggravating inflammation and exacerbating insulin resistance. Moreover, the presence of NF-*κ*B in different tissues may trigger distinct signals to mediate the complex manifestations of overnutrition-induced diseases. Therefore the activation of the NF-*κ*B may be considered not only a key mechanism for the development of insulin resistance but also an important contributor for metabolic dysfunction and the development of nutrition-overload induced complications. Seen in this light, blockade of NF-*κ*B activity would be imperative to maintain cellular homeostasis and adequate physiological function in obesity (Figure 2). Moreover, dysfunctional metabolism due to excessive inflammation may lead to premature aging in obesity.

Although obesity is escalating in all population groups, a causal relationship between obesity and premature aging has been postulated for years. The molecular mechanisms involved in obesity-induced aging are only beginning to be unraveled now. Recent evidence suggests that obesity accelerates the aging of adipose tissue due to increased formation of reactive oxygen species in fat cells and shortened telomeres which ultimately results in activation of the p53 tumor suppressor, inflammation, and the promotion of insulin resistance and hypertension [192, 193]. Therefore obesity may be considered a chronic stress factor that creates a pathphysiological milieu that may ultimately compromise the metabolic system. Overnutrition-induced chronic stress offsets the balance between metabolic and immune functions and contributes to the development of visceral obesity, T2D; and the metabolic syndrome. Moreover, obesity-induced proinflammatory cytokines from the adipose tissue may act as an additional chronic stimulus for stimulation of other stress-related pathways including the hypothalamic-pituitary-adrenal axis [194], creating a vicious cycle between metabolic and immune responses during nutrient overload. Accordingly, obesity-induced stress has been reported to impair the systemic metabolic homeostasis [37]. Conversely, stress has been linked to the development of visceral obesity [177]. Generally, stress is characterized by elevated levels of glucocorticoid, a hormone implicated in the development and differentiation of preadipocytes [195]. Reports indicate that glucocorticoids regulate the expression of the stress-related enzyme 11b-hydroxysteroid dehydrogenase (11b-HSD). This enzyme has dual function as it converts inactive cortisone to active 11b-HSD1 or, alternatively, the conversion of cortisol to inactive 11b-HSD2 [196]. 11b-HSD1 induces stress and has been linked to the development of obesity and insulin resistance [197–199]. Supportive of this notion are experiments demonstrating that knocking-out 11b-HSD1 suppressed the development of obesity and insulin resistance, whereas overexpression of 11b-HSD1 led to the development of obesity [197–199]. Consistently, the activity of 11b-HSD is elevated in obsessed humans [200, 201]. Of more-importance and even more intriguing is the finding that the ability to regulate 11b-HSD is lost in T2D patients, whereas it is compromised in nondiabetic obsessed individuals [201]. These findings highlight the central role of glucocorticoids in regulating metabolism via 11b-HSD, and suggest that the regulation of 11b-HSD is a dynamic process that becomes gradually impaired or even completely compromised as the severity of the obesity worsens when it progresses to metabolic syndrome and/or T2D. Interestingly motifs for glucocorticoid-responsive element are present in the HO-1 promoter [160, 161]. Whether this is indicative of a role of the HO system in the modulation of glucocorticoid-induced stress and/or involvement in glucocorticoid-induced regulation of 11b-HSD remains the subject of future investigations. However, this hypothesis is particularly attractive because stress is linked to the development of visceral obesity [177], a condition in which glucocorticoids play a key role in the development and differentiation of preadipocytes [195]. Interestingly, the HO system has been shown to suppress visceral and subcutaneous obesity [40, 111–113, 202]. Therefore, the HO-mediated suppression of visceral and subcutaneous obesity when combined to other cytoprotective effects of the HO system such as the attenuation of NF-*κ*B activity [41–44, 47, 83, 84, 203] may constitute a protective shield against insulin resistance, obesity, and other nutrition-overload related complication (Figure 2). Accordingly, the presence of motifs for glucocorticoid-responsive elements and binding sites for many substances including sites for inflammatory/oxidative transcription factors like NF-*κ*B, AP-1 and AP-2 in the HO-1 promoter [160, 161] suggest that the HO system may be playing a more important role in metabolism that previously thought. 

Although obesity was first described as low-grade inflammation more than a decade ago, it is only recently that obesity-induced increase of macrophage infiltration of adipose tissue and elevated number of classically activated macrophages or M1-type has been associated with obsessed individuals [204–206]. It is becoming increasingly clear that the adipose tissue is infiltrated by macrophages that trigger inflammatory events in obesity [207, 208]. Moreover, the dramatic shift of the pool of macrophages from the alternatively-activated M2-type to the classically-activated M1-type results in changes in secreted cytokines from predominantly anti-inflammatory (M2-type) to proinflammatory (M1-type) in obese conditions, although the exact mechanism for this shift remains largely unclear [204–206]. Since alternatively activated macrophages have a beneficial role in regulating nutrient homeostasis, an increase of alternatively-activated M2-type might be a useful strategy for treating insulin resistant T2D [205]. Given that PPAR*γ* is necessary for the maturation of alternatively activated macrophages [205], and PPAR*γ* is a transcription factor that regulates adipogenesis, insulin sensitization and inflammation, the potentiation of PPAR*γ*-signalling would be beneficial in obesity [209–213].

 Interestingly emerging evidence indicates that the HO system suppresses different inflammatory events including macrophage infiltration [54, 63, 111, 202, 214] and potentiate insulin sensitivity and glucose metabolism in obesity [40, 111, 113] in a similar way as PPAR*γ* [209–213]. Accordingly, cross-talk between PPAR*γ* and the HO system has been reported [215]. Moreover, analysis of human HO-1 promoter using a combination of transient transfection experiments, mutational analysis, and gel shift assays has demonstrated the direct transcriptional regulation of HO-1 by PPAR*γ* and PPAR*α* [215]. Consistently, the notion that HO-1 is a PPAR target gene [216, 217] has been further strengthened by the observation that HO-1 enhances the levels of PPAR*γ* protein expression and activity [218]. On the other hand, PPAR*γ* has also been shown to induce HO-1 [217]. Therefore, a mutual reciprocal stimulatory relationship between PPAR*γ* and the HO system can be envisioned [217, 218] and coordination of this synergistic interaction between these two systems may constitute a novel and potent strategy to combat obesity-induced complications and other related problems like T2D, insulin resistance, hypertension, and metabolic syndrome. Given the recent findings that HO inducers enhance insulin sensitivity and improve glucose metabolism in different insulin resistant rats strains including ZDF and GK [219, 220] and obese mouse [40, 111, 113], it is tempting to speculate that the HO-mediated suppression of macrophage infiltration constitutes not only an important anti-inflammatory mechanism to limit tissue insult in hypertension but also a mechanism that could be explored to improve insulin sensitivity and glucose metabolism in obsessed individuals with insulin resistance and overt T2D.

### 3.4. The HO System, Oxidative Stress, and Insulin Signalling

Many studies have underscored the role of oxidative stress in insulin resistance [174, 221–223]. Reactive oxygen species are produced by the electron transport system in mitochondrial respiration and are increased in conditions associated with enhanced oxidation of energy substrate such as glucose and free-fatty acids. Reports indicate that factors that increase oxidative stress like hyperglycemia, free-fatty acids and adipokines contribute to insulin resistance [174, 222]. Although the exact mechanism of insulin resistance is not fully understood, recent data suggest the implication of oxidative stress in the pathogenesis of multiple forms of insulin resistance [174, 221–223]. Thus, there is a general consensus that elevated oxidative stress unleash the cascade of events that impairs insulin-signalling [174, 222, 223]. As such, insulin resistance may be regarded as a state of increased exposure to reactive oxygen species [174, 222], and thus strategies that concomitantly reduce oxidative stress, glucose/insulin intolerance and lower blood pressure may improve glucose metabolism. Generally, the skeletal muscles accounts for 65%–90% of the clearance of glucose clearance [140]. Under healthy conditions, the vascular actions of insulin stimulate the production of NO from the endothelium leading to vasodilation and increased blood flow to skeletal muscles that enhance glucose-uptake [224]. However, in hypertensive conditions, elevated levels of superoxide quenche NO by forming peroxynitrite [225], that subsequently oxidizes arachidonic acid to generate 8-isoprostane, a potent vasoconstrictor which may decrease skeletal muscle blood flow, and thus reduce glucose-uptake. 

Although many studies support the link between hypertension and insulin resistance, the underlying mechanisms are not completely understood. However, CO from the HO system and NO may be implicated because these vasoactive gases are important not only as a vasodilators, but also in the regulation of insulin signaling [45, 46, 226–230]. Recent evidence indicates that insulin stimulates the production of NO [45, 46, 226], and thus insulin may regulate blood pressure via the NO pathway. The binding and subsequent activation of IRS-1 and IRS-2 by insulin triggers a cascade of events that ultimately lead to activation of PI3K and protein kinase (PKB) or Akt. In healthy subjects, both P13K and Akt activate endothelial NO synthase to generate NO [231, 232] and thus promote vasodilation. However, in insulin-resistant conditions, oxidative stress impairs the activation of P13K/Akt-signaling resulting in impaired vasorelaxation [232–234]. Similarly, TNF*α* impairs vasorelaxation by inhibiting the P13K/Akt-signaling [233, 235]. The P13K/Akt-signaling is important for glucose transport and is involved in the translocation of GLUT4 to the cell membrane [232]. However, in hypertensive subjects, these cascades of events may be impaired, and so insulin-stimulated NO may be insufficient [232] leading to reduced vasorelaxation, decreased blood to skeletal muscles, and impaired translocation of GLUT4. Thus, hypertension and insulin resistance may compromise endothelial function and cause overt T2D.

Since GLUT4 and effective dilation of skeletal muscle and are largely responsible for glucose disposal, reduced GLUT4 translocation and impaired skeletal muscle dilation would result in reduced removal of glucose, leading to hyperglycemia, hyperinsulinemia, and eventually insulin resistance [232, 236]. Alternatively, diminished action of insulin and the resultant hyperglycemia may result in the accumulation of advanced glycation end-products (AGE) and this would increase oxidative/inflammatory events [237–239], which in turn would further increase the production of AGE, and thus creating a vicious cycle that potentiates the oxidative destruction of beta-cells in both T1D and T2D [237, 240–242]. Moreover, increased oxidative stress and AGE may lead to DNA damage, the activation of NF-*κ*B, and deranged transcription [235], all of which will accentuate cell damage. Therefore the progressive loss of beta-cell function and the corresponding decline of insulin production reported in TD1 and TD2 could be attributed, at least in part to oxidative stress [243, 244]. Accordingly, the maintenance of specialized islet architecture and the regulation of beta-cell number by antioxidants and antiapoptotic agents may be important for the preservation of intact pancreatic structure to safeguard the insulin-producing capability of beta-cells. Interestingly, our recent studies indicate that upregulating the HO system enhances GLUT4 expression and improves glucose metabolism [41–44, 47, 48]. On the other hand, the P13K/Akt-signaling may also regulate vascular contractility and blood pressure homeostasis by modulating calcium ion transport [232, 234, 245]. Moreover, insulin triggers vasodilatation by inhibiting voltage-gated calcium influx [232, 234]. Similarly, glucose transport and glucose-6-phosphate synthesis have been reported to reduce smooth muscle vascular resistance by enhancing calcium efflux [232, 234]. The P13K/Akt-signaling and glucose transport may be blunted in the pathophysiological conditions like insulin resistance and hypertension [232, 234]. The dysfunctional P13K/Akt-signaling coupled to reduced calcium efflux may result in elevated vascular resistance in insulin resistant diabetes and hypertensive conditions [232, 234]. Therefore oxidative stress, impaired glucose transport and utilization, and reduced NO production are amongst the contributing factors of hypertension and these factors may also lead to the development of insulin resistance [232, 233, 246]. 

From the above mentioned studies, it could be envisaged that elevated vascular resistance may constitute a common denominator in hypertension and insulin resistant diabetes, and strategies like HO inducers that enhance vascular relaxation [228, 229] and improves glucose metabolism [38–52] may constitute an alternative approach to simultaneously combat hypertension and insulin resistance in patients symptomatic with these comorbid conditions. However, given that many insulin resistant patients are normotensive, further studies are needed to fully characterize the P13K/Akt-signaling and calcium efflux in hypertension and insulin resistance. Given the close association between the P13K/Akt-signaling and the HO system [247–251], further exploration of these pathways may lead to better understanding of the multifaceted interaction between the HO system and the P13K/Akt-signalling and the development of novel strategies against hypertension and insulin resistance.

## 4. Concluding Remarks

Obesity, insulin resistant T2D, and many related cardiometabolic complications share a metabolic milieu characterized by elevated inflammatory/oxidative insults. While inflammation-induced insulin resistance is increasing in parallel with the epidemic of obesity and metabolic syndrome, there are additional unrelated mechanisms associated with the polygenic conditions of insulin resistance, T2D, and cardiometabolic complications that create a great challenge for future therapeutic modalities. With the polygenic nature of these conditions, treatment strategies should not be limited to monogenic targets. Interestingly, emerging data have underscored the role of the HO system in insulin sensitivity and cellular metabolism. The HO system has been shown to suppress visceral and subcutaneous obesity [40, 111–113, 202], potentiating the antioxidant status in cells and abating oxidative/inflammatory mediators including 8-isoprostane JNK AP-1 and AP-2 [41–44, 47, 83, 84, 203]. These qualities, in combination to the HO-mediated attenuation of NF-*κ*B activity [41–44, 47, 83, 84, 203] may constitute a protective shield against insulin resistance, obesity, and other nutrition-overload-related complications. Moreover, the presence of motifs for glucocorticoid-responsive elements and binding sites for many substances including sites for inflammatory/oxidative transcription factors like NF-*κ*B, AP-1 and AP-2 in the HO-1 promoter [160, 161], suggest that the HO system may be playing a more important role in the regulation of cellular metabolism. 

Finally, the mutual reciprocal stimulatory relationship between PPAR*γ* and the HO system may be explored in the design of novel remedies. The coordination of this synergistic interaction may constitute a novel approach that could be explored in the search of more-effective and potent strategies against the polygenic conditions of insulin resistance, T2D, and cardiometabolic complications.

## Abbreviations

(AP-1): Activating protein-1
(AP-2): Activating protein-2
(AMPK): Adenosine monophosphate-activated protein kinase
(AGE): Advanced glycation end-products
(CO): Carbon monoxide
(cAMP): Cyclic adenosine monophosphate
(cGMP): Cyclic guanosine monophosphate
(JNK): c-Jun-N-terminal kinase
(DNA): Deoxyribonucleic acid
(DOCA): Deoxycorticosterone-acetate
(GK): Goto-Kakizaki rats
(GLUT4): Glucose-transporter-4
(IKK*β*): Inhibitor of nuclear factor kappa B kinase subunit beta
(IRS-1): Insulin receptor substrate-1
(IL-6): Interleukin
(IL-1*β*): Interleukin-1 beta
(IPGTT): Intraperitoneal glucose-tolerance
(IPITT): Intraperitoneal insulin-tolerance
(HO): Heme oxygenase
(HOMA): Homeostasis model of insulin resistance
(11b-HSD): 11b-hydroxysteroid dehydrogenase
(LPS): Lipopolysaccharide endotoxin
(NO): Nitric oxide
(NF-*κ*B): Nuclear-factor kappaB
(PPAR*γ*): Peroxisome proliferator-activated receptors gamma
(PKB or Akt): Protein kinase
(PI3K): Phosphatidylinositol 3-phosphate kinase
(RAS): Renin-angiotensin-aldosterone system
(RNA): Ribonucleic acid
(STZ): Streptozotocin
(TLR4): Toll-like receptor
(T1D): Type-1 diabetes
(T2D): Type-2 diabetes
(TNF-*α*): Tumour-necrosis factor-alpha
(ZDF): Zucker diabetic fatty rats.
